# The flaccid life

**Published:** 2009

**Authors:** Mahesh Pundlik Kate

**Affiliations:** Department of Neurology, Sri Chitra Tirunal Institute for Medical Sciences and Technology, Trivandrum, India. E-mail: maheshkate@yahoo.co.in

With tingling in the fingers it did start,
In front of the eyes, my life was torn apart.

Slipped buckled I became bed bound,
Doctor you are asking again my breath counts!

Is it treatable? What is the course?
Or is it going to get bitter-more-worse.

Oh dear, spinal or cranial, the nerve-roots are on fire,
It's your body defense, has gone hay wire.

Virus, vaccine, bacteria, or tumor somewhere,
Playing with you to cause damage elsewhere.

Absence of fluctuating vitals keeps me happy,
Blowing the candle and breath counts comes handy and snappy.

Pheresis or globulins are much the same,
It's for worsening or discharge to be written early at your name.

Toiling nightingales will lift your spirit,
With their devotion to serve, health is going to make it.

Physio with boost of morale, I can see the future,
May require few months it's all in the nature.

